# Multidisciplinary Treatment of Inguinoscrotal Sarcomas: Analysis of 39 Cases Treated by Surgical Approach †

**DOI:** 10.3390/cancers18050876

**Published:** 2026-03-09

**Authors:** Roger Homs Samsó, Lorena Cambeiro Cabré, Sandra González Abós, Mireia Solans Solerdelcoll, Katarina Majercakova, Ana Sebio García, Isidre Gracia Alegria, Manuel Fernández Garrido, Antonio Moral Duarte, José Antonio González López

**Affiliations:** 1 Department of General Surgery, Hospital de la Santa Creu i Sant Pau, 08041 Barcelona, Spainlcambeiro@santpau.cat (L.C.C.);; 2 Department of General Surgery, Hospital Universitari Mútua de Terrassa, 08221 Terrassa, Spain; 3 Department of Radiation Oncology, Hospital de la Santa Creu i Sant Pau, 08041 Barcelona, Spain; 4 Department of Medical Oncology, Hospital de la Santa Creu i Sant Pau, 08041 Barcelona, Spain; 5 Department of Orthopaedics and Trauma Surgery, Hospital de la Santa Creu i Sant Pau, 08041 Barcelona, Spain; 6 Department of Plastic and Reconstructive Surgery, Hospital Clínic Barcelona, 08036 Barcelona, Spain

**Keywords:** inguinoscrotal sarcoma, liposarcoma, multidisciplinary approach

## Abstract

Background: Inguinoscrotal sarcomas are a rare sarcoma subtype. The treatment of choice is radical inguinal orchiectomy with wide local resection of the surrounding soft tissues. However, consensus regarding prognostic factors is lacking. We present our experience at a referral sarcoma center concerning the management, oncologic results, and prognostic factors pertaining to this disease. Methods: We conducted a retrospective analysis of patients who underwent surgery for inguinoscrotal sarcomas between 2005 and 2023 at a sarcoma referral hospital. Results: The study included 39 patients. The most frequent histology was liposarcoma. Seven patients required surgical reconstruction with a microvascularized free flap. Four patients presented major postoperative complications. Mean follow-up was 46 months. Overall survival rates were 97.4%, 81.7%, and 64.8% at one, three, and five years. High-grade tumors were correlated with worse overall and disease-free survival. Conclusions: The chance finding of a sarcoma in the inguinal region poses a diagnostic and therapeutic dilemma when considering options for treatment with curative intent. Vascular and muscle resection followed by vascular and/or free flap reconstruction may be necessary to achieve complete surgical resections; therefore, a multidisciplinary approach is needed. A preoperative biopsy should be performed to establish the histological grade, which may be the main prognostic factor.

## Simple Summary

Inguinoscrotal sarcomas are extremely rare tumors. The treatment of choice is complete resection during the first surgery. A multidisciplinary approach is needed as a radical resection may necessitate vascular or free flap reconstruction for limb preservation. The aim of this study was to improve knowledge of this disease by presenting our experience regarding its management, oncologic results, and prognostic factors. A retrospective analysis was conducted on 39 patients treated surgically at our sarcoma referral hospital.. Histological grade has been confirmed as one of the main prognostic factors, being associated with worse overall and disease-free survival.

## 1. Introduction

Inguinoscrotal sarcomas have a low incidence (0.3–0.5 cases per million) [1]. They constitute only 2% of all soft-tissue sarcomas and 1% to 2% of malignant tumors of the genitourinary tract [2].

The rarity of these tumors and their low clinical suspicion makes diagnosis difficult, likely leading to delays in treatment. The typical presentation of inguinoscrotal sarcomas is a unilateral inguinal tumor or scrotal mass [3,4,5]. It may or may not be painful and is occasionally accompanied by a hydrocele. When the inguinal mass is small, it is commonly mistaken for an inguinal hernia or lipoma and surgery is performed without radiological testing or prior biopsy [6,7].

In adults, more than 75% of these lesions arise from the spermatic cord [1,8]. Due to the low incidence of these inguinal tumors, consensus is lacking regarding prognostic factors, although most authors appear to consider low-grade tumor and complete resection to be factors associated with a favorable outcome [1,9].

The current standard therapeutic approach for these tumors is radical orchiectomy with wide local resection of the surrounding soft tissues [1,10]. In patients with positive margins after the initial surgery, a re-resection to widen margins is believed to be associated with a lower risk of recurrence by improving local disease control [11]. When aggressive surgery involving critical structures of the inguinal region is needed to obtain negative surgical margins, a multidisciplinary approach including tissue and vascular reconstruction should be performed [12]. This multidisciplinary approach not only aims to solve immediate problems arising from oncological resection but also to avoid possible complications in the short, medium, and long term in patients who undergo postoperative radiotherapy, such as wound dehiscence, lymphorrhea, lymphangitis or lower limb lymphedema.

To avoid possible incomplete resection, preoperative distinction between malignant paratesticular tumors and benign inguinoscrotal lesions is mandatory. However, in most clinical cases in the literature, these tumors are diagnosed after histopathologic analysis after a first surgery which is performed without prior biopsy [1,6,7]. This results in an incomplete surgical procedure and poorer oncologic outcomes.

To decrease the risk of local recurrence after surgery, radiotherapy in the preoperative or postoperative setting may be feasible, but few studies regarding this issue are available to date [13,14,15]. Radiotherapy may be especially indicated for large, bulky lesions, high-grade tumors, R1–R2 resections, or local recurrences. When interpolating data from studies of sarcomas of the extremities, in preoperative radiotherapy, the volume irradiated is lower than postoperative radiotherapy and the dose is 45–50 Gy. However, patients have a higher risk of wound healing complications than those who receive radiotherapy after surgery. In postoperative radiotherapy, the treatment field is wider, and the dose is higher (50–70 Gy) [16].

In relation to complications, the use of adjuvant or neoadjuvant radiotherapy seems to increase the risk of wound dehiscence and risk of fibrosis [16]. In such cases it is mandatory to achieve good reconstruction of local soft tissue defects. Local or microsurgical free flaps should be considered in patients with large resections (>10 cm), in those previously treated with radiotherapy, and in those with vascular reconstruction in order to ensure correct soft tissue coverage. In these patients, radical tumor resection, including the radiate superficial tissue and adequate soft tissue reconstruction with microsurgical free flaps may be the best option to avoid complications [12].

Regarding organs at risk, intensity-modulated radiation therapy (IMRT) allows for better preservation of organs at risk, and no high-grade toxicity has been reported to date [17]. However, further studies are needed to obtain more information on the impact and ideal sequencing of this type of treatment.

As a referral sarcoma hospital, the aim of this study was to describe our experience in the surgical treatment of inguinoscrotal sarcomas based on postoperative results and long-term survival.

## 2. Materials and Methods

### 2.1. Patient Sample

We retrospectively analyzed 39 patients diagnosed with inguinoscrotal sarcoma who underwent surgical treatment between January 2005 and December 2023 at Hospital de la Santa Creu i Sant Pau, a National Reference Centre for Sarcomas in Spain.

Tumor nomenclature was updated according to the current WHO Classification of Tumors of Soft Tissue. Specific AJCC/TNM staging was not uniformly applied due to the retrospective nature of the study; therefore, tumors were categorized based on size (<5 cm vs. ≥5 cm) and metastatic status (M0 vs. M1). Regarding treatment policy, neoadjuvant radiotherapy (RT) was considered for large or high-grade tumors, while adjuvant RT was generally indicated for R1/R2 margins or high-grade lesions. Systemic chemotherapy was reserved for high-grade (grade 3) tumors, metastatic disease, or selected high-risk cases discussed in the multidisciplinary committee.

We reviewed histopathological analysis and only patients with primary inguinoscrotal sarcomas were selected. No patients were lost to follow-up. Data collected were age, tumor laterality, date of surgery, surgical technique, histopathologic subtype, tumor characteristics (grade and stage), location and date of recurrence additional treatment and follow-up.

The indication for surgical resection was established by a multidisciplinary mesenchymal tumors committee in accordance with the hospital’s protocol and based on the patient’s baseline characteristics and radiological findings. Primary treatment consisted of radical excision of the primary tumor or revision surgery and margin widening. Adjuvant treatment was discussed by a multidisciplinary sarcoma committee.

### 2.2. Histological Grade

The histological grade of the tumor was assigned following the Federation Nationale des Centres de Lutte Contre le Cancer (FNCLCC) [18] based on histopathological type and tumor differentiation, necrosis, and mitotic activity. Tumors were classified as either grade 1–2 or grade 3.

### 2.3. Follow-Up

All patients were routinely followed up with CT or MRI scans of the surgical site every three months during the first postoperative year and every six months thereafter. Local recurrence (LR) was strictly defined as a recurrence confined to the primary tumor bed or regional inguinoscrotal soft tissues in the absence of distant metastases. Disease recurrence was defined as the first clinical or radiological finding of local or distant disease after resection of the primary tumor. Mortality was associated with the disease in patients who had uncontrolled recurrent disease at the time of death.

### 2.4. Statistical Analysis

Distributions were summarized using frequencies, medians, and ranges. Overall survival rates were estimated using the Kaplan–Meier method and the log-rank test. Given the low number of events, multivariate analysis was not performed. Any *p*-value < 0.05 was considered statistically significant. All analyses were performed using IBM SPSS Statistics version 25^®^ software.

## 3. Results

### 3.1. Patient and Tumor Characteristics

Thirty-nine patients (eight women and thirty-one men) were included. Mean age was 60 years [24–90] at the time of diagnosis of the primary tumor. In thirty-one patients (31/39, 79.5%), the clinical symptoms were associated with the disease. The first manifestation of the disease in one patient was paraneoplastic thrombotic syndrome in the form of pulmonary thromboembolism. Most patients were referred to our center due to suspicion of inguinal hernia, lipoma, or an undiagnosed inguinal mass. Eight patients were previously misdiagnosed as suspected lipoma, and the first symptom in one patient was a suspected incarcerated inguinal hernia. In 2 patients (2/39, 5.1%) the diagnosis was an incidental radiological finding (Table 1).

Twenty-two patients underwent a preoperative biopsy (22/39, 56.4%). Of the seventeen patients who underwent primary surgery at our hospital, fourteen had undergone a preoperative biopsy (14/17, 82.3%). Meanwhile, among the twenty-two patients whose initial surgery was performed at another center, only eight had a prior biopsy (8/22, 36.4%). This difference is statistically significant (*p* < 0.001).

The most frequent histology findings were liposarcoma (18/39, 46.2%) and leiomyosarcoma (6/39, 15.4%). The remaining anatomopathological findings are described in Table 2. There were four cases of Dermatofibrosarcoma protuberans, which were included in this series because they were deep-seated in the inguinoscrotal region and therefore required wide local resections. Other tumor characteristics are summarized in Table 3.

The four cases of Dermatofibrosarcoma protuberans showed no fibrosarcomatous transformation; these were included as they were deep-seated and required wide local resections, although they represent a biologically distinct entity from conventional soft-tissue sarcomas.

### 3.2. Surgical Procedures and Immediate Postoperative Outcomes

As specified before, twenty-two patients (22/39, 56.4%) had undergone initial surgery in another hospital and were then referred to our institution. Seventeen of the 22 underwent a second intervention. In 8 cases, surgery was completed by a wide resection, and in the remaining 9 patients, margins were widened. Four patients underwent wide resection at the first surgery.

Of the 17 patients who underwent initial surgery at our center, 3 required reinterventions to widen margins. One of the three had had an erroneous initial diagnosis of adenopathy. Taken together, the surgical procedures performed in our hospital were wide resection in 25 patients, 17 as initial surgery and 8 to complete resection after the initial surgery. Nine patients underwent margin widening surgery.

Seven patients (7/39, 17.9%) required immediate surgical reconstruction with a microvascularized free flap and two required common femoral vein reconstruction (with no associated postoperative complications).

Regarding the postoperative evolution, 82.1% (32/39) of patients presented no complications, 7.7% (3/39) presented minor complications (grade 1 or 2 according to the Clavien–Dindo classification), and 10.3% (4/39) presented major complications.

Three patients had surgical wound infection requiring reoperation for debridement and subsequently required a microsurgical free flap to cover the resulting defect. Only one patient required admission to the Intensive Care Unit (this being due to respiratory failure caused by exacerbation of chronic obstructive pulmonary disease).

There were no deaths in the first 30 postoperative days.

All surgical procedures were performed in a similar manner and included extensive resection to obtain negative margins, including the deep inguinal orifice and the soft tissue of the inguinal canal with ligation of the inferior epigastric vessels. Surgery did not vary in relation to histological subtype. Reconstructive surgery was performed only in patients who had vascular involvement of the tumor.

Surgery was performed by means of a multidisciplinary approach involving 4 surgical teams (from general surgery, orthopedic surgery, plastic surgery, and vascular surgery). Only one surgical team was needed in most cases of primary surgery. In cases of margin widening or local recurrence resection that required a reconstructive procedure, combined surgery including general, plastic, and vascular surgeons was performed.

### 3.3. Surgical Margins

After the initial surgery, R0 margins were achieved in nineteen patients (19/39, 48.7%), whereas twelve patients presented R1 margins (12/39, 30.8%) and eight patients presented R2 margins (8/39, 20.5%). All the patients with an R2 margin had initially undergone surgery at another center, and a second surgical procedure was performed at our institution.

No statistically significant differences were observed among the three groups regarding recurrence, local recurrence, overall survival o disease-free survival.

When the results obtained after the second surgery performed to widen margins were pooled, R0 margins twenty-six patients presented R0 margins (26/39, 66.6%) and the remaining thirteen had R1 margins (13/39, 33.3%).

### 3.4. Neoadjuvant and Adjuvant Therapy

Six patients (6/39, 15.4%) received neoadjuvant therapy, four patients (4/39, 10.5%) received chemotherapy alone, and one patient (1/39, 2.6%) received a combination of radiotherapy and chemotherapy. Only one patient (1/39, 2.6%) received neoadjuvant radiotherapy in monotherapy.

A total of 21 patients received adjuvant treatment (21/39, 53.8%), 6 received chemotherapy only (6/39, 15.4%), 12 received exclusive radiotherapy (12/39, 30.8%), and 3 received a combination of chemotherapy and radiotherapy (3/39, 7.7%). Recurrence occurred in 13 of the 21 patients who received adjuvant treatment (61.9%), and in 4 of the 18 patients who did not receive this treatment (22.2%).

Regarding radiotherapy, patients received standard doses for inguinal sarcomas (typically 50–70 Gy). No major complications or severe wound healing issues directly attributable to RT were recorded in this series. However, detailed data on specific RT techniques (2D/3D/IMRT) and exact dose ranges were not available for all historical cases.

Patients who received adjuvant treatment presented lower overall (27.6% vs. 90%, *p* = 0.001) and disease-free survival (21.2% vs. 62.9%, *p* = 0.005).

Patients with histological grade 3 tumors received more frequently adjuvant therapy than those with grade 1–2 tumors (85.7% vs. 27.7%, *p* = 0.001). No statistically significant differences were observed between the two groups regarding tumor size, definitive pathological findings, resection margin status, or the presence of metastases at the time of diagnosis. Table 4 details all the results distributed between the group that received adjuvant therapy and the group that did not.

### 3.5. Follow up, Recurrence, and Survival

The mean follow-up was 53.41 months with a median of 43 months [3–179]. No patients were lost to follow-up. Recurrence occurred in seventeen patients (17/39, 43.6%), six patients experienced local recurrence (6/39, 15.4%), five patients developed distant metastatic disease (5/39, 12.8%), and six patients had both local and distant recurrence (6/39, 15.4%).

Among patients with local recurrence (12/39, 30.8%), no statistically significant differences were observed in relation to histological grade (25.9% vs. 58.3%, *p* = 0.101), margin status, the use of adjuvant local radiotherapy, or the institution where the initial surgery was performed. These findings are summarized in Table 5.

**Figure 1:**
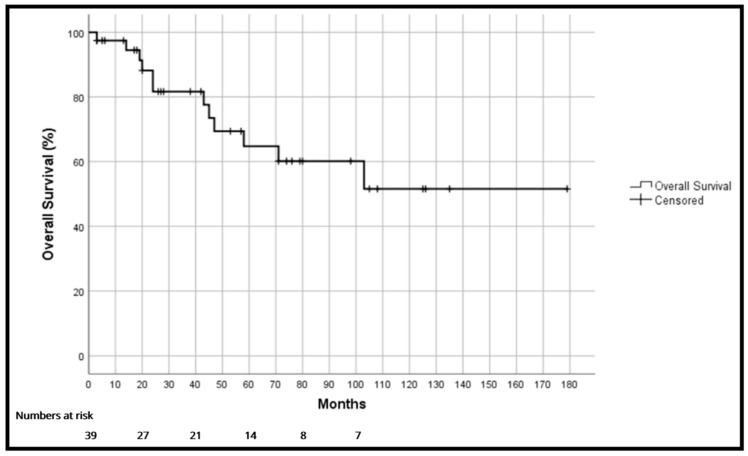
Overall survival.

**Figure 2:**
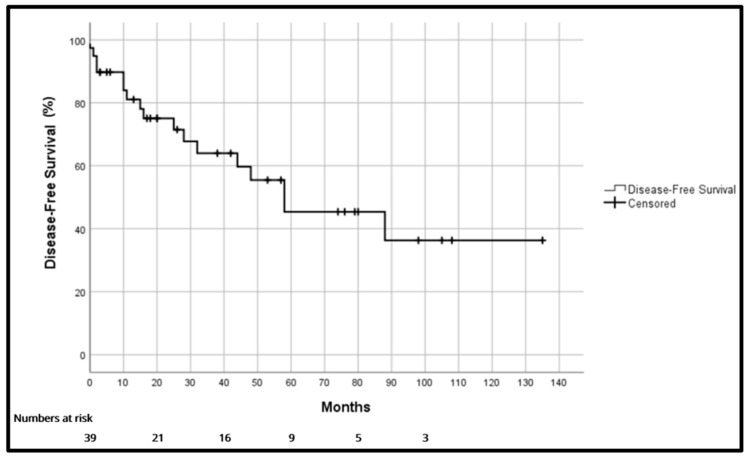
Disease-free survival.

Overall survival (OS) was 97.4%, 81.7% and 64.8% at one, three and five years, respectively (Figure 1). Disease-free survival (DFS) was 81.1%, 64% and 45.4% at one, three and five years, respectively (Figure 2). Median disease-free survival was 58 months [17–99]. Local recurrence-free survival was 86.5%, 71.4% and 61.1% at one, three and five years, respectively.

Of the seventeen patients who presented recurrence, ten of them (10/17, 58.8%) received surgical treatment. Of the twelve patients who presented local recurrence, eight underwent salvage surgery (66.7%). Some of these procedures required highly complex surgery, including sigmoidectomy in one patient. Extensive resections requiring plastic surgery reconstruction were needed in three patients (one requiring a free flap). Two patients needed major vascular resections.

### 3.6. Prognostic Factors

Due to the small sample size, multivariate analyses of prognostic factors in relation to overall survival and disease-free survival were not performed.

Table 6 and Table 7 therefore summarize the univariate analyses of potential prognostic factors associated with overall survival and disease-free survival.

#### Histological Grades

**Figure 3:**
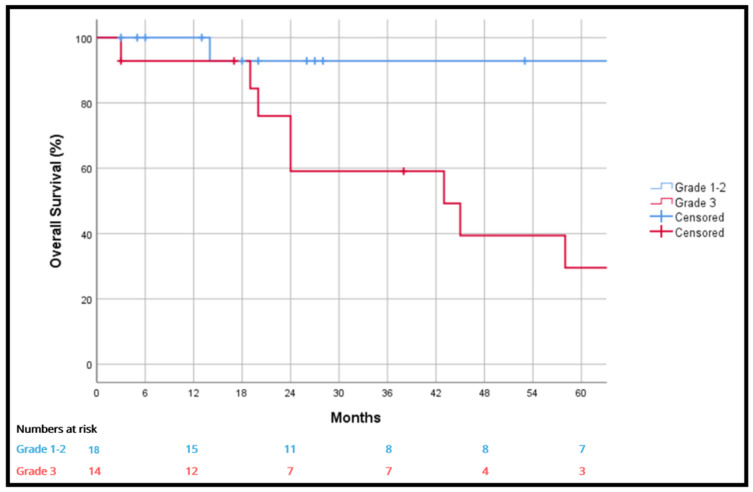
Overall survival according to grade 1–2 vs. grade 3.

**Figure 4:**
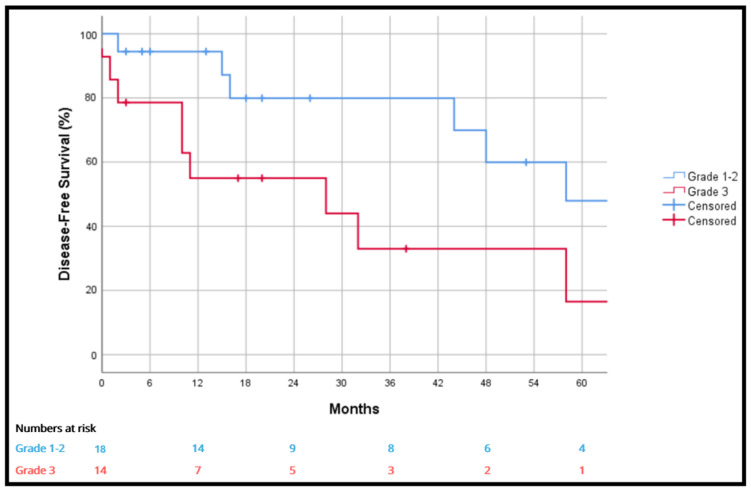
Disease-free survival according to grade 1–2 vs. grade 3.

At one, three and five years, grade 3 tumors were significantly associated with worse overall survival than grade 1–2 tumors (92.9% vs. 100%, 59.1% vs. 92.9% and 29.5% vs. 92.9%; *p* = 0.006) (Figure 3) and disease-free survival at one, three and five years (55% vs. 94.4%, 33% vs. 79.9% and 16.5% vs. 47.9%; *p* = 0.019) (Figure 4). The liposarcoma histology subgroup had a better five-year survival rate than other tumors (77% vs. 52.1%), but without statistical significance (*p* = 0.811).

## 4. Discussion

Inguinoscrotal sarcomas are very rare malignant neoplasms of the inguinal and paratesticular region. Publications regarding oncologic outcomes, adjuvant therapies and prognostic factors are few, and they consist predominantly of case series [19,20,21,22]. Several cohorts describe few patients over decades; for example, a single-center study over a 20-year period collected only 22 inguinoscrotal sarcomas [23], while Chowdry et al. describe one of the largest single-center series, with 34 patients over a 46-year period [24]. Regarding longer series, Khan et al. presented a study conducted in a tertiary referral sarcoma center, with a sample of 108 patients [25]. More recently, the French Sarcoma Group presented one of the largest series, including a total of 224 patients [26].

Compared to extremity or retroperitoneal soft-tissue sarcomas (STS), inguinoscrotal sarcomas present unique surgical challenges due to the proximity of critical structures and the limited anatomical space for wide margins, often requiring complex multidisciplinary reconstruction.

The chance finding of a sarcoma in the scrotum or inguinal region often poses a surgical dilemma when considering options for complete resection [27,28]. The common presentation as lipoma or inguinal hernia indicates the difficulties faced in diagnosing and studying these tumors, leading to initial misdiagnosis. A total of 56.4% of patients in our series (22 of 39) were initially explored and treated at other hospitals before being referred to our hospital for further treatment. We found that the most common histology was liposarcoma (46.2%), followed by leiomyosarcoma (15.4%)—results that correspond to those described in the literature [1,26].

The treatment of choice for sarcomas located in soft tissues is complete surgical resection with negative margins (R0), and surgical technique does not vary according to histologic subtype. However, there is no consensus on second revision surgery in patients with marginal surgery, although it is recommended in those with positive surgical margins (R1). Some authors report that positive surgical margins significantly increase the risk of local recurrence, in addition to increasing the risk of distant metastases and disease-related mortality [1,11,25,26,29].

In patients with locally advanced disease, extensive resections (including muscle and/or vascular tissue) may be mandatory, which means that in order to save the limb it may be necessary to use vascular and/or free flap reconstruction techniques. A multidisciplinary approach should be considered for any intervention with these characteristics, including the support of specialties such as plastic surgery, orthopedic surgery and vascular surgery [12]. Although no differences in survival were found in our group of patients, local recurrence seems to be lower in those who underwent aggressive resections. In those patients who received pre- or post-surgical radiotherapy, immediate and complete local soft tissue reconstruction with free flaps was associated with a low number of local complications such as wound dehiscence or infection, with lymphedema being the main postoperative local complication.

Rodriguez et al. analyzed the SEER (Surveillance, Epidemiology, and End Results Program) cancer registry in the United States, compiling a cohort of 362 patients, which is the largest we have found in the literature. This review concludes that undifferentiated tumor grade, advanced stage, positive lymph nodes, and positive histology for histiocytoma or leiomyosarcoma would be independent predictors of worse disease-specific survival [1].

In our series, the overall survival rates found were 97.4%, 81.7% and 64.8% at one, three and five years, respectively. These results are similar to those we have found in the literature, but the lack of larger series and their heterogeneity makes it difficult to compare these results [24,25,26,27].

In our cohort, tumor histological grade had a significant impact on overall survival. Grade 1–2 tumors had a longer overall survival compared to those with grade 3 tumors. Our series has a high rate of high-grade tumors (43.8%) compared to previous series published in the literature, although we describe a similar survival rate [1,24,25,26].

We have also found differences in disease-free survival when comparing histological grade. Patients with a diagnosis of liposarcoma presented a higher overall survival at five years, although not statistically significantly (*p* = 0.811). Achard et al. described similar results in their series [26].

Regarding other prognostic factors, the presence of metastases at diagnosis was the only factor significantly associated with poorer overall and disease-free survival. However, as only two patients in the cohort presented with metastases at diagnosis, these findings, while clinically plausible, should be interpreted with caution.

Due to the limited sample size and the low number of events, only univariate analyses were performed to assess prognostic factors. Multivariate analyses were not conducted, as including multiple variables would risk overfitting and produce unstable estimates. Therefore, the results should be interpreted with caution, and future studies with larger cohorts are needed to confirm these findings.

No other prognostic factors with statistical significance were found in our series, but again, this may be due to its limited sample size.

In our series, patients who received adjuvant treatment had a higher recurrence rate and lower overall and disease-free survival. Nevertheless, these data are most likely biased by the fact that patients who received adjuvant treatment had more unfavorable tumors with a higher histologic grade or greater aggressiveness at the time of diagnosis (as specified in Section 3.3 of the Results), leading to confounding by indication. There is no evidence in the literature concerning the role that adjuvant treatment may play in treating this type of tumor, nor its effect in preventing locoregional recurrence or improving the survival [16,30]. Further studies should be conducted to determine its importance, as well as to identify which patients may benefit the most.

These findings should be regarded as exploratory and non-causal. They are likely driven by confoundin by indication, as patients selected for adjuvant therapy typically had more unfavorable baseline features such as higher histological grade or more aggressive disease at diagnosis.

Khan et al. [25] compared the oncological results among patients treated primarily in a sarcoma referral center versus those who had an initial surgery in another hospital and were then referred to their center to complete the excision. Despite a lower R0 resection rate in the initial surgery, those patients who received the second procedure did not present higher risk of recurrence. Although differences in the time intervals required to achieve treatment targets were not correlated with the risk of recurrence, their series showed that patients who required a second surgical intervention experienced a higher rate of postoperative complications. Although these findings were not observed in our study, we strongly recommend the mandatory referral of these patients to sarcoma referral centers, to optimize the diagnostic process as well as to improve postoperative results.

The local recurrence rate in our series was higher than that reported by Khan et al. (30.8% vs. 10.2%). Unlike previous reports, we did not identify any prognostic factors significantly associated with local recurrence. This discrepancy may be partly explained by the limited sample size of our study. Moreover, the higher proportion of high-grade tumors in our cohort compared with the other series may have contributed to the increased local recurrence rate.

In their series, higher tumor grade and size were correlated with a worse distant metastasis free survival and overall survival [25]. These results are similar to those obtained in our series and indicate that the tumor grade is probably the main prognostic factor.

In the full series, marginal resection (R1) correlated with an inferior local recurrence free survival; however, this association did not reach statistical significance in our study.

To our knowledge, our study has one of the largest and most homogeneous cohorts of patients diagnosed by inguinoscrotal sarcoma, as it is based on the experience of a single center and a dedicated multidisciplinary team. Our findings regarding tumor characteristics and prognostic factors are consistent with those previously published.

Nevertheless, the limited sample size represents a clear limitation of this study. Being a retrospective study, potential selection bias and unmeasured confounding cannot be excluded. Reliance on univariate analyses may increase susceptibility to potential confounding factors, such as neoadjuvant or adjuvant therapy, the institution where the initial intervention was performed, resection margin status, and tumor histology. Finally, the heterogeneity of the patient population and the tumor characteristics may limit the generalizability of the results.

## 5. Conclusions

Correct preoperative diagnosis and optimal surgery are vital to achieving satisfactory results and avoiding the need for second surgery to widen the margins. This second procedure is often more complex and has a higher rate of complications.

Our series contributes to increasing our knowledge of this rare entity by providing a large number of cases and establishing the histological grade as the main prognostic factor. Furthermore, we demonstrate the need for a multidisciplinary approach to the surgical treatment of these tumors in order to achieve their complete resection. Further studies involving larger series are needed to establish, with greater evidence, the main prognostic factors, as well as to develop adjuvant therapies to improve the oncologic outcomes.

## Figures and Tables

**Table 1:** Demographic characteristics and clinical manifestations.

Baseline Characteristics		n (%)
Median age (years)		60
Age distribution (years)	<50	5 (12.8)
	50–70	27 (69.2)
	>70	7 (18)
Gender	Male	31 (79.5)
	Female	8 (20.5)
ASA *	I	6 (15.4)
	II	20 (51.3)
	III	13 (33.3)
	IV	0 (0)
Clinical manifestations	Symptoms	33 (84.6)
	· Inguinal lump	32
	>Suspected lipoma	8
	>Suspected incarcerated hernia	1
	>Inguinal mass	23
	· Pulmonary thromboembolism	1
	Incidental radiological findings	2 (5.1)
	Incidental anatomopathological findings	4 (10.3)

* ASA: Preoperative classification of the American Society of Anesthesiology.

**Table 2:** Pathological anatomy.

Definitive Pathological Anatomy	n (%)
Liposarcoma	18 (46.2)
Leiomyosarcoma	6 (15.4)
Dermatofibrosarcoma protuberans	4 (10.3)
Solitary fibrous tumor	2 (5.1)
Synovial sarcoma	2 (5.1)
Undifferentiated Pleomorphic sarcoma	2 (5.1)
Ewing’s sarcoma	1 (2.6)
Rhabdomyosarcoma	1 (2.6)
Low-grade Fibromyxoid sarcoma	1 (2.6)
Spindle cell sarcoma	1 (2.6)
Extraskeletal osteosarcoma	1 (2.6)

**Table 3:** Tumor characteristics.

		n (%)
Mean size (mm; DE)		66.6 (62.4)
Histological grade	Grade 1	9 (23.1)
	Grade 2	9 (23.1)
	Grade 3	14 (35.9)
	Missing	7 (17.9)
Metastases status at the time of diagnosis	M0	37 (94.9)
	M1	2 (5.1)

**Table 4:** Tumor characteristics according to adjuvant therapy status.

		Non Adjuvant Therapy	Adjuvant Therapy *
		18	21
**Histological grade** **n (%)**	Grade 1–2	13 (72.2)	5 (23.8)
	Grade 3	2 (11.1)	12 (57.1)
	Missing	3 (1.7)	4 (19.1)
**Mean size** **mm (SD)**		58.94 (67.4)	73.10 (58.6)
**Pathological findings** **n (%)**	Liposarcoma	8 (44.4)	10 (47.6)
	Leiomyosarcoma	4 (22.2)	2 (9.5)
	Other	6 (33.3)	9 (42.9)
**Margin status** **n (%)**	R0	13 (72.2)	13 (61.9)
	R1	5 (23.8)	8 (38.1)
**Metastases at the time of** **diagnosis** **n (%)**	No	18 (100)	19 (90.5)
	Yes	0 (0)	2 (9.5)

* Includes chemotherapy or radiotherapy alone, and combination of radiotherapy and chemotherapy.

**Table 5:** Local recurrence.

		No Local Recurrence	Local Recurrence
		27	12
**Histological grade** **n (%)**	Grade 1–2	14 (51.9)	4 (33.3)
	Grade 3	7 (25.9)	7 (58.3)
	Missing	6 (22.2)	1 (8.3)
**Margin status** **n (%)**	R0	19 (70.3)	7 (58.3)
	R1	8 (29.7)	5 (41.7)
**Initial surgery** **n (%)**	Referral center	12 (44.4)	5 (41.7)
	Other	15 (55.6)	7 (58.3)
**Radiotherapy**	Yes	17 (63)	6 (50)
	No	10 (37)	6 (50)

**Table 6:** Univariate Cox regressions analysis for overall survival.

		HR	95% CI	*p*-Value
**Age at diagnosis (years)**	≥60 vs. <60	0.89	0.29–2.76	0.836
**Tumor size**	≥5 cm vs. <5 cm	1.01	0.32–3.21	0.984
**Histological grade**	Grade 3 vs. Grade 1–2	6.67	1.43–31.13	0.016
**Margin status**	R1 vs. R0	1.77	0.57–5.51	0.324
**Initial surgery**	Referral center vs. Other	0.66	0.21–2.07	0.474
**Metastases at diagnosis**	M1 vs. M0	7.92	1.59–39.51	0.012
**Adjuvant therapy**	Yes vs. No	14.33	1.82–113.02	0.012

**Abbreviations**: HR, Hazard ratio; CI, confidence interval. **Note**: Univariate Cox proportional hazard models were used.

**Table 7:** Univariate Cox regressions analysis for disease-free survival.

		HR	95% CI	*p*-Value
**Age at diagnosis (years)**	≥60 vs. <60	0.67	0.24–1.82	0.429
**Tumor size**	≥5 cm vs. <5 cm	0.53	0.201–1.399	0.200
**Histological grade**	Grade 3 vs. Grade 1–2	3.32	1.19–9.29	0.022
**Margin status**	R1 vs. R0	1.49	0.57–3.93	0.417
**Initial surgery**	Referral center vs. Other	0.87	0.335–2.268	0.778
**Metastases at diagnosis**	M1 vs. M0	25.63	3.51–187.19	0.001
**Adjuvant therapy**	Yes vs. No	4.33	1.40–13.41	0.011
**Adjuvant radiotherapy**	Yes vs. no	1.92	0.738–4.99	0.181

**Abbreviations**: HR, Hazard ratio; CI, confidence interval. **Note**: Univariate Cox proportional hazard models were used.

## Data Availability

The data presented in this study are available on request from the corresponding author due to it involves medical information of the patients included in the study.
